# FTH1 overexpression using a dCasRx translation enhancement system protects the kidney from calcium oxalate crystal-induced injury

**DOI:** 10.1186/s11658-024-00582-w

**Published:** 2024-05-07

**Authors:** Ziqi He, Caitao Dong, Tianbao Song, Jiawei Zhou, Tao Xu, Ruyuan He, Sheng Li

**Affiliations:** 1 Department of Urology, Renmin Hospital of Wuhan University, Wuhan, 430060 Hubei People’s Republic of China; 2 Department of Urology, Zhongnan Hospital of Wuhan University, Wuhan, 430071 Hubei People’s Republic of China; 3 Department of Urology, Huanggang Central Hospital of Yangtze University, Huanggang, 438000 Hubei People’s Republic of China; 4 Department of Thoracic Surgery, Renmin Hospital of Wuhan University, Wuhan, 430060 Hubei People’s Republic of China

**Keywords:** CRISPR–Cas system, dCasRx, eIF4GI, Gene regulation, Protein translation, Nephrolithiasis, FTH1, Ferroptosis

## Abstract

The engineered clustered regularly interspaced short palindromic repeats (CRISPR)–CRISPR-associated protein (Cas) system is currently widely applied in genetic editing and transcriptional regulation. The catalytically inactivated CasRx (dCasRx) has the ability to selectively focus on the mRNA coding region without disrupting transcription and translation, opening up new avenues for research on RNA modification and protein translation control. This research utilized dCasRx to create a translation-enhancement system for mammals called dCasRx-eIF4GI, which combined eukaryotic translation initiation factor 4G (eIF4GI) to boost translation levels of the target gene by recruiting ribosomes, without affecting mRNA levels, ultimately increasing translation levels of different endogenous proteins. Due to the small size of dCasRx, the dCasRx-eIF4GI translation enhancement system was integrated into a single viral vector, thus optimizing the delivery and transfection efficiency in subsequent applications. Previous studies reported that ferroptosis, mediated by calcium oxalate (CaOx) crystals, significantly promotes stone formation. In order to further validate its developmental potential, it was applied to a kidney stone model in vitro and in vivo. The manipulation of the ferroptosis regulatory gene FTH1 through single-guide RNA (sgRNA) resulted in a notable increase in FTH1 protein levels without affecting its mRNA levels. This ultimately prevented intracellular ferroptosis and protected against cell damage and renal impairment caused by CaOx crystals. Taken together, this study preliminarily validated the effectiveness and application prospects of the dCasRx-eIF4GI translation enhancement system in mammalian cell-based disease models, providing novel insights and a universal tool platform for protein translation research and future therapeutic approaches for nephrolithiasis.

**Supplementary Information:**

The online version contains supplementary material available at 10.1186/s11658-024-00582-w.

## Introduction

Protein translation is a multistep biological process encompassing initiation, elongation, termination, and ribosomal recycling. Cytoplasmic ribosomes are recruited to mRNA, scanning its 5′ untranslated region (5′ UTR) to identify the translation initiation codon for subsequent reactions. Therefore, translation initiation is a crucial rate-limiting step [1]. Eukaryotic translation initiation factor 4G (eIF4G) is an important protein that regulates the beginning of translation in eukaryotes by bringing 40S ribosomal subunits to mRNA templates or internal ribosomal entry site (IRES) elements [2]. Moreover, eIF4G, a substantial modular protein, is able to promote the creation of the eukaryotic translation initiation factor 4F (eIF4F) through its interaction with the cap-binding protein (eIF4E) and RNA helicase (eIF4A) [3]. The eIF4G family has two subtypes, namely eIF4GI and eIF4GII, and earlier studies have documented that the former is the predominant form in HeLa cells, constituting approximately 85% of the total eIF4F complex and serving as a functional core module protein [4]. It is worth mentioning that proteins combined with eIF4GI can enhance translation by increasing ribosome recruitment without raising mRNA levels. Due to the lack of extensive research on tools to initiate mRNA translation in mammalian cells, eIF4GI fusion proteins were chosen as modified components to activate translation in mammalian cells.

The clustered regularly interspaced short palindromic repeats (CRISPR)–CRISPR-associated protein (Cas) system can be categorized into class 1 and class 2 CRISPR–Cas systems. The effector architecture of the class 1 CRISPR–Cas system is intricate, necessitating the coordinated involvement of multiple Cas proteins to carry out the specific function of the system. Conversely, the effector of the class 2 CRISPR–Cas system consists of a solitary Cas protein encompassing a substantial domain. The organizational structure of the loci in the class 2 system is comparatively more streamlined and standardized than that of the class 1 CRISPR–Cas system, thereby exhibiting enhanced transformative potential [5, 6]. Among these systems, the class 2 CRISPR–Cas system employs a solitary effector protein (Cas9, Cas12, and Cas13), which can be categorized into three distinct groups based on the variation in effector proteins. Notably, Cas9 and Cas12a have found extensive application in the realm of genetic engineering [7]. Notably, the CRISPR–Cas9 system can not only precisely target and edit gene sequences but also simultaneously activate or suppress the transcription of endogenous genes. With advances in the technology platform, the CRISPR–Cas9 system can be used for both DNA and RNA regulation [8, 9]. The Cas13 system, identified as CRISPR–Cas VI-D, has been defined as a new family capable of guiding and targeting RNA. Cas13 enzymes, including Cas13a, Cas13b, Cas13c, and Cas13d, possess dual RNA cleavage activities, rendering them suitable for precise RNA editing and the detection of RNA fluctuations in biological processes [10].

Cas13d is the smallest subtype of the Cas13 family, with RfxCas13d (CasRx) identified as the smallest known Cas13 enzyme exhibiting excellent knockdown performance [10–12]. Remarkably, when CasRx is deactivated catalytically (dCasRx), it has the ability to target genes in a specific manner without causing notable changes in their mRNA and protein levels, suggesting that it can effectively focus on the mRNA coding region without disrupting the translation process. This creates new opportunities for research on RNA modification and the regulation of protein translation. Contrary to past methods focused on boosting mRNA levels of target genes, translation is the ultimate stage of gene expression that directly impacts gene expression intensity [13]. Hence, utilizing the CRISPR–dCasRx platform for translation regulation editing technology could be a promising strategy in attaining increased efficiency and stable gene overexpression.

To achieve this goal, a translation enhancement tool platform for mammalian cells was pioneered based on the CRISPR–dCasRx system. In mammalian cells, the translation initiation of certain coding genes was activated by combining dCasRx-single-guide RNA (sgRNA) with the eIF4GI translation enhancement element to increase protein production. By meticulous planning and fine-tuning, the dCasRx translation enhancement system was able to consistently produce and operate efficiently in mammalian cells with just one lentivirus (LV) or adenoassociated virus (AAV) vector. Additionally, this system can enhance the translation levels of various endogenous proteins in mammalian cells. To further explore the applicability of the dCasRx translation enhancement system under different physiological and pathological conditions, it was applied in a kidney stone disease model.

Nephrolithiasis, commonly referred to as kidney stones, is a prevalent urological disease characterized by high incidence and recurrence rates, as well as high treatment costs, thereby posing a significant burden on healthcare institutions globally [14, 15]. Twin and genealogical studies also have demonstrated that nephrolithiasis has been influenced by a strong genetic factor. Urinary characteristics associated with kidney stone formation have been found to be highly inherited [16, 17]. As is well documented, calcium oxalate (CaOx) stones are the most frequently encountered type of kidney stones, and the oversaturation of CaOx crystals in urine has been identified as an independent risk factor for stone formation [18, 19]. And numerous studies have demonstrated that the formation of CaOx kidney stone highly likely originated from the injured renal tubular epithelial cells stimulated by abnormal urine, including hypercalciuria, hyperoxaluria, and the oversaturation of CaOx crystals [20, 21]. Our long-term study on the impact of excessive CaOx crystals in the development of CaOx kidney stones has shown that CaOx crystal-induced ferroptosis in HK-2 cells is a significant factor in causing cell damage from calcium oxalate crystals and is crucial in facilitating stone formation [22]. Following ferroptosis, GPX4 biosynthesis levels decrease, leading to the inability to inhibit the lipid peroxidation process [23]. Simultaneously, ferritin heavy chain 1 (FTH1), containing an iron oxidase center, is a major functional subunit that stores iron as a “mineral” within ferritin. Its autophagic degradation may lead to the excessive accumulation of cellular Fe^2+^ and play a fundamental role in activating ferroptosis [24]. The degradation of FTH1 is a central pathway for intracellular iron overload and greatly enhanced susceptibility to ferroptosis [25, 26]. In this research, the dCasRx translation enhancement system was utilized to simultaneously target the FTH1 gene, boost the protein translation of FTH1 in HK-2 cells and kidney stone mouse models, elevate FTH1 protein expression, effectively reduce ferroptosis, and protect against cell-crystal damage caused by CaOx crystals, ultimately preventing kidney stone formation. Altogether, our observations signal that the translation enhancement system has high development and application potential.

In ferroptosis-related studies, most agonists and inhibitors that target ferroptosis control the extent of ferroptosis by either stimulating or blocking lipid peroxidation levels [23]. Limited studies have reported interventions targeting iron or Fe^2+^ accumulation [27]. Thus, this study proposed an intervention strategy based on the dCasRx translation enhancement system to modulate Fe^2+^ accumulation to indirectly regulate ferroptosis. Herein, a translation enhancement tool for mammalian genes was successfully constructed, offering a novel platform for translation research. The method was simultaneously used in a CaOx kidney stone disease model, yielding initial findings and suggesting a potential molecular treatment strategy involving RNA editing control for future management of ferroptosis-related conditions and kidney stones.

## Methods

### Plasmid development and assembly process

We created a new dual-vector system to control gene expression by chemically synthesizing the genetic sequence that codes for the dCasRx enzyme. This engineered sequence was then integrated into a vector under the control of the CMV promoter. To improve performance, the eIF4GI domain located at the end of the vector was also integrated into this structure. To facilitate nuclear import, nuclear localization signals (NLS) were appended to both ends of the resultant fusion protein's coding sequence. Conversely, for components operating in the cytoplasm, the incorporation of NLS tags was deemed unnecessary. The complementary DNA strand for the guide RNA (sgRNA) was cloned into another vector harboring the U6 promoter, enabling the precise targeting of desired mRNA molecules. In the construction of a streamlined single-vector system for translation control, superfluous elements such as fluorescent markers and NLS motifs were omitted. Afterward, the sequences encoding the fusion protein and sgRNA were combined into a single vector, resulting in the development of a small dCasRx-eIF4GI-sgRNA plasmid. The fusion protein and sgRNA were expressed under the control of the CMV and U6 promoters, respectively. High-scoring sequences targeting the specified protein were selected to refine the sgRNA design using the online tool found at https://cas13design.nygenome.org.Detailed sequence information is delineated in the Additional file 1**: **Table S1, Additional file 2: Table S2, Additional file 3: Table S3.

### Cell culture and experimental model development

In our research, HK-2 cells obtained from the Cell Bank of the Chinese Academy of Sciences (SCSP-511, Shanghai, China) were grown in ideal settings at a temperature of 37 °C and a CO_2_ concentration of 5%. The cells were cultured in Dulbecco’s modified Eagle medium/nutrient mixture F-12 (F-12) medium supplemented with 10% fetal bovine serum. To replicate the harm caused by CaOx crystals, HK-2 cells were exposed to a 1 mM CaOx solution for 24 h. Upon achieving 30–50% confluency, cells were transfected using a 50:50 mixture of the dual-vector system (DV) transfection agent and Lentivirus (LV), or a single-vector system (SV) transfection-lentivirus mixture at a multiplicity of infection (MOI) of 50, as per the manufacturer’s protocols. After transfection, the medium was changed within 8–12 h to eliminate the transfection mixture. Selection with 5 μg/mL puromycin over 2 weeks facilitated the establishment of stable cell lines.

### 4D-Label free quantitation (4D-LFQ) proteomic analysis

Samples were retrieved from −80 ℃ storage and mixed with the lysis buffer at a ratio of 1:4. Protein levels were measured with the Bicinchoninic Acid Assay (BCA) test to ensure consistent protein quantities in all samples for future procedures. The protein of each sample was enzymatically digested. Protein analysis was conducted using liquid chromatography–mass spectrometry (LC–MS). Data analysis was performed with MaxQuant software against the Homo_sapiens_9606 database, incorporating both a reverse database and common contaminant filters to ensure accuracy. Proteins were classified according to GO annotations into biological processes, cellular components, and molecular functions, with enrichment analysis used to detect significant categories. A two-tailed Fisher's exact test was conducted to compare the presence of differentially expressed proteins in these categories with the dataset, with *P*-values < 0.05 deemed as statistically significant.

### Animals

The animal experiment was approved by Laboratory Animal Welfare and Ethics Committee of Renmin Hospital of Wuhan University (issue no. 20230705F). This study involved twelve male C57 mice, within the age bracket of 6–8 weeks, with their weights ranging from 24 to 28 g, sourced from the Disease Control and Prevention Centers of Hubei Province. These mice were housed under strict specific pathogen-free conditions. The humidity levels were consistently maintained at a range of 40–70%, while the temperature was kept around 22 ± 2 °C. They experienced a light–dark cycle lasting 12 h each and were given free access to both food and water throughout the study. During the experiment, the mice were randomly split into two groups, with six mice in each group, one acting as the control group and the other as the SV-FTH1 stone model group. An adenoaassociated virus (AAV) vector encapsulating the dCasRx-eIF4GI-sgRNA construct was prepared for administration. While under anesthesia, the kidney of each mouse was exposed and the ureter was clamped to block flow, then the prepared SV-FTH1 AAV solution (1 × 10^11^ μg/per mouse) was injected into the renal pelvis. After 4 weeks from the injection, the levels of FTH1 expression were evaluated. Mice were given daily intraperitoneal injections of glyoxylic acid (80 mg/kg, obtained from Sigma-Aldrich; Merck KGaA) for 14 days to induce kidney stone formation. After the last glyoxylic acid treatment, mice were anesthetized with a pentobarbital injection (50 mg/kg) to obtain kidney tissues and blood samples, and then euthanized with a higher dose of pentobarbital (100 mg/kg) in a humane manner.

### Quantitative real-time reverse transcription PCR (qRT–PCR)

Total RNA was extracted from cell cultures and mouse kidney tissues using Trizol (15596026, Thermo Fisher) following the manufacturer’s instructions to determine the gene expression levels. The Hifair^®^ III 1st Strand cDNA Synthesis Kit (gDNA digester plus; 11139ES60, Yeasen, China) was used to conduct complementary DNA (cDNA) synthesis using 2 μg of RNA as a template. Quantification of gene expression was conducted using the LightCycler 480 system from Roche Diagnostics in the USA, utilizing the Hieff UNICON^®^ Universal Blue qPCR SYBR Green Master Mix (11184ES08) from Yeasen in China. Gene expression was standardized based on GAPDH levels using the 2^−△△Ct^ method to quantify changes in gene expression. Each sample was subjected to three independent qRT–PCR analyses. The primer sequences employed in this study will be shown as follows:

GAPDH, 5′-GTCTCCTCTGACTTCAACAGCG-3′ (forward) and 5′-ACCACCCTGTTGCTGTAGCCAA-3′ (reverse);

FTH1, 5′-TGAAGCTGCAGAACCAACGAGG-3′ (forward) and 5′-GCACACTCCATTGCATTCAGCC-3′ (reverse);

GPX4, 5′-GTAAACTACACTCAGCTCGTCGA-3′ (forward) and 5′-TTGATCTCTTCGTTACTCCCTGG-3′ (reverse);

SIRT1, 5′-TAGACACGCTGGAACAGGTTGC-3′ (forward) and 5′-CTCCTCGTACAGCTTCACAGTC-3′ (reverse);

BNIP3L, 5′-TGTGGAAATGCACACCAGCAGG-3′ (forward) and 5′-CTACTGGACCAGTCTGATACCC-3′ (reverse).

### Western blotting (WB)

After conducting experimental procedures, proteins were isolated from samples of cellular and renal tissue from mice using a cold RIPA lysis buffer (G2002, Servicebio, China). The concentration of the extracted proteins was determined through BCA protein assays (PC0020, Solarbio Science and Technology, China). Following this, each sample was treated with 30 μg of protein and then underwent electrophoresis on 10–12% sodium dodecyl sulfate–polyacrylamide gel electrophoresis (SDS–PAGE) gels. The proteins were subsequently transferred onto Immun-Blot polyvinylidene fluoride (PVDF) membranes (1620177, BIO-RAD, USA).Membrane blocking was accomplished by applying a rapid blocking buffer (PS108P, Epizyme, China) for 10 min, then proceeding with multiple washes in Tris-buffered saline containing Tween 20 (TBST).Afterward, the membranes were left to incubate overnight at 4 °C with primary antibodies that targeted FTH1 (ab65080, 1:1000, Abcam, UK), GPX4 (A1933, 1:1000, ABclonal, China), CD71 (A5865, 1:1,000, ABclonal, China), ASCL4 (22401-1-AP, 1:4000, Proteintech, USA), SIRT1 (13161-1-AP, 1:2,000, Proteintech, USA), NIX (A24803, 1:1000, ABclonal, China), Tubulin (11224-1-AP, 1:10,000, Proteintech, USA), and GAPDH (10494-1-AP, 1:20,000, Proteintech, USA). After additional TBST washes, the membranes were then exposed to suitable secondary antibodies at room temperature for a duration of 1 h. Protein bands were observed with an Odyssey dual-color infrared laser imager from LI-COR in the USA, and the intensities of the bands were measured using ImageJ software version 1.51j8.

### Immunofluorescence assay

Cells were grown in 24-well plates until they were about 70% confluent before undergoing the specified treatment protocols for immunofluorescence analysis. Following that, the cells were washed thrice using phosphate-buffered saline (PBS) and then treated with 4% paraformaldehyde (P0099, Beyotime, China) for a duration of 15 min. After three more PBS washes, the cells were then blocked with 5% BSA (ST2249, Beyotime, China) at room temperature, followed by another three PBS washes. Anti-FTH1 primary antibodies (ab65080, 1:100, Abcam, UK) were used to incubate the cells overnight at 4 °C. Following PBS rinsing, the cells were then exposed to a secondary antibody at room temperature for a duration of 1 h. Coverslips were finally sealed with antifade mounting medium containing DAPI (P0131, Beyotime, China). The fluorescence signal was evaluated using a BX53 fluorescence microscope (Olympus, Japan).

### Cell count kit-8 (CCK-8) assay

The viability of cells was assessed in 96-well dishes by performing the CCK-8 test from Biosharp (BS350B, China). Following the intervention, 100 µl of CCK-8 solution were added to every well, and incubation was carried out for 2 h at 37 ℃. The absorbance was recorded at 450 nm using an EnSight microplate reader (PerkinElmer, USA).

### Assessment of antioxidant and oxidative stress markers

We measured the total antioxidant capacity (T-AOC), superoxide dismutase (SOD), catalase (CAT), and Malondialdehyde (MDA) levels to assess oxidative stress and antioxidant capacity. Specific assay kits from Nanjing Jiancheng (T-AOC, A015-2-1, China; SOD, A001-3-2, China; MDA, A003-4-1, China) and Beyotime (CAT, S0051, China) were used according to the manufacturer’s instructions. The absorbance values were determined using an EnSight microplate reader.

### Reactive oxygen species (ROS) detection

Intracellular ROS levels were measured using the DCFH-DA ROS Assay Kit from Dojindo Laboratories (R252, Japan), known for its high sensitivity. DCFH-DA permeates into cells where it is oxidized, emitting green fluorescence. Following treatment, the cell culture medium was removed and the cells were exposed to the DCFH-DA solution that had been prepared, incubating at 37 °C for 30 min in the absence of light. Following the incubation period, the cells were washed two times using Hank’s Balanced Salt Solution (HBSS) to eliminate any remaining dye. The fluorescence indicating levels of ROS was observed using an Olympus IX71 inverted fluorescence microscope from Japan at a magnification of ×400.

### Intracellular iron quantification

For quantifying intracellular iron levels, the Iron Assay Kit (DOJINDO, I291, Japan) was employed. After the treatments, mouse kidney tissue and HK-2 cell samples were lysed using the iron detection buffer provided. Following the guidelines provided by the kit, the absorbance of the reaction mix was assessed at 593 nm with a microplate reader. Furthermore, the FerroOrange assay (DOJINDO, F374, Japan) facilitated the visual assessment of intracellular Fe^2+^ levels. The presence of Fe^2+^ within the cells was detected using a 400× magnification inverted fluorescence microscope (Olympus, IX71, Japan).

### Lipid peroxidation detection

To quantify lipid peroxidation, 15,000 cells per well were plated in 24-well plates and cultured for 24 h prior to exposure to CaOx (1 mM) for an additional 24 h. Lipid peroxidation was measured utilizing the Liperfluo reagent (Dojindo Laboratories, L248). Treated cells were incubated with 10 μM Liperfluo for 1 h at 37 °C to stain them. The fluorescence signal was subsequently quantified using a flow cytometer (Beckman Coulter, CytoFlex, USA), with data analysis performed via FlowJo 10.6.2 software. Additionally, the Lipid Peroxidation Probe -BDP 581/591 C11 assay (Dojindo Laboratories, L267) was employed for further lipid peroxidation assessment. Visualization of lipid peroxidation was achieved through an inverted fluorescence microscope (Olympus, IX71, Japan).

### Transmission electron microscopy (TEM)

The morphological changes in mitochondria were examined through transmission electron microscopy (TEM), following standardized procedures. After intervening, HK-2 cells and kidney tissue samples were fixed then subjected to a dehydration process using ethanol and acetone, each for 15 min. Subsequent to dehydration, sections of 60 nm thickness were prepared, placed on copper grids (200 mesh) and examined under a TEM microscope (JEOL, Tokyo, Japan). This method allowed for the detailed observation of mitochondrial structures and any morphological alterations induced by the treatments.

### Serum creatinine (CRE) and blood urea nitrogen (BUN) measurement

After the mice received treatment, blood was collected from their veins for examination. An automated clinical chemistry analyzer was used to quantitatively measure the levels of creatinine and blood urea nitrogen, offering valuable information about kidney function.

### Histological analysis

Immunohistochemical staining was used to examine the levels of renal GPX4 and ASCL4 expression. Kidneys were excised, fixed in 4% paraformaldehyde, and embedded in paraffin. Tissue sections were then dewaxed in xylene, rehydrated through an ethanol gradient, and subjected to antigen retrieval. Primary antibodies against GPX4 (A1933, 1:100, ABclonal, China) and ASCL4 (A14439, 1:100, ABclonal, China) were used to incubate sections overnight at 4 °C. After incubating with the primary antibody, the sections were then exposed to secondary antibodies for 1 h at room temperature. Following a series of washing, staining, counterstaining, dehydrating, and mounting steps, the slides were observed using a fluorescence microscope (BX53, Olympus) to analyze protein expression qualitatively.

### Calcium salt staining

For calcium salt detection, kidney sections were dewaxed, rehydrated, and treated with silver nitrate under ultraviolet light for 10 min. After washing and treatment with hyposulfite solution, sections were counterstained with hematoxylin and eosin (Solarbio, G1120, China) and examined under a fluorescence microscope (Olympus, BX53).

### Statistical analysis

Experimental data are presented as mean ± standard error of the mean (SEM) from at least three independent experiments. Statistical comparisons were performed using GraphPad Prism version 8.0 (GraphPad Software, Inc.), employing one-way ANOVA followed by Bonferroni’s post hoc test. Significance was established at *P* < 0.05.

## Results

### dCasRx translation enhancement system: preliminary design and functional validation

**Fig. 1:**
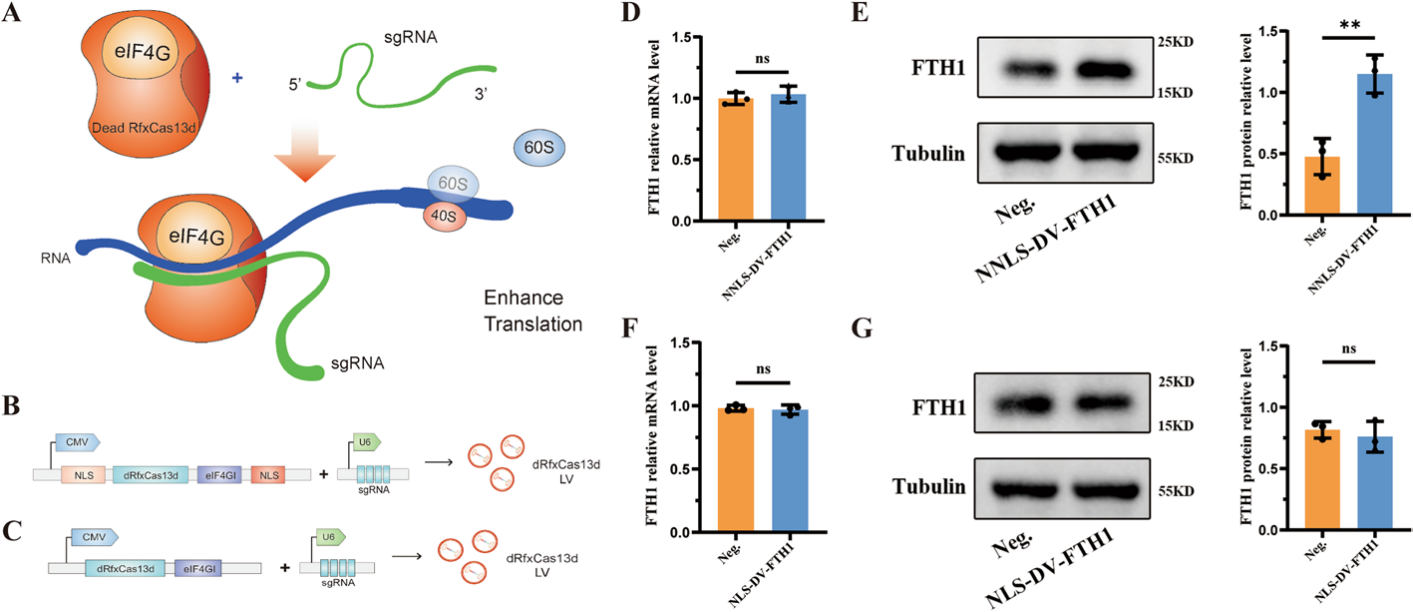
Preliminary design and functional validation of dCasRx translation enhancement system. **A** The eIF4GI was selected to fuse with dead CasRx. Then the CRISPR-dCasRx-eIF4GI increases translation of proteins based on sgRNAs by targeting sequences that bind specific RNAs. **B** The dCasRx-eIF4GI fusion protein, the NLS and sgRNA complementary DNA sequence of related genes were cloned into the plasmids containing CMV promoter and U6 promoter, respectively, for packaging into Lentiviral vectors. **C** To package into lentiviral vectors, the dCasRx-eIF4GI fusion protein and its complementary DNA sequence, sgRNA, were cloned into plasmids containing the CMV promoter and U6 promoter, respectively. **D** After constructing the no NLS CRISPR-dCasRx-eIF4GI tool targeting FTH1 (NNLS-DV-FTH1), the mRNA expression level of FTH1 were detected using qRT–PCR analysis. **E** The protein expression of FTH1 were verified using western blotting (WB) and the bar graph shows the relative expression level of FTH1. **F** After constructing the NLS CRISPR-dCasRx-eIF4GI tool targeting FTH1 (NLS-DV-FTH1), the mRNA expression level of FTH1 were detected using qRT–PCR analysis. **G** The protein expression of FTH1 were verified using WB and the bar graph shows the relative expression level of FTH1. Data are presented as the means ± SEM from three independent experiments. *P < 0.05, **P < 0.01, ***P < 0.001, ****P < 0.0001, and ns, no significant difference, represents *P* > 0.05

eIF4G is a large modular protein that can form a translation initiation complex, eIF4F, by binding with eIF4E and eIF4A. This complex constitutes the minimal core functional structure supporting translation initiation. It also serves as a crucial molecular bridge between the mRNA 5′ end and the ribosomal subunit, recruiting the 40S ribosomal subunit to the cap structure at the mRNA 5′ end. Then, the 40S subunit recruits the 60S subunit to initiate peptide synthesis, a vital step in translation initiation. As an essential functional fusion protein in eIF4F, eIF4G plays a key regulatory role in cap-dependent translation initiation in eukaryotic organisms. In the present study, the dCasRx was selected as the binding component for the target RNA and eIF4GI as the translation enhancement component. eIF4GI was fused to the C-terminus of dCasRx to form a translation regulatory domain. At the same time, the dCasRx-eIF4GI fusion protein vector and sgRNA vector were driven by CMV and U6 promoters, respectively. The translation enhancement effect of the target RNA was achieved through the synergistic action of the dual vectors (Fig. 1A). Earlier studies have insinuated that CasRx exhibits stronger RNA targeting specificity following the localization to the cell nucleus compared with its localization in the cytoplasm. However, translation largely occurs in the cytoplasm. Consequently, the translation enhancement tool was divided into a nuclear localization signal (NLS) group and a no NLS group by inserting or omitting NLS signals at both ends of the dCasRx-eIF4GI protein. Two sets of fusion protein LV vectors and sgRNA-FTH1 LV vectors (MOI 50:50) were constructed and transfected into HK-2 cells (Fig. 1B and C). Through stable transfection screening, the mRNA and protein levels of FTH1 were detected using RT-qPCR and western blot analysis. The transcription levels of FTH1 were comparable in both groups (Fig. 1D and F). On the other hand, compared with the negative control group, the protein level of FTH1 was significantly higher in the no NLS group (Fig. 1E), while it was unchanged in the NLS group (Fig. 1G). These results collectively suggest that the translation enhancement effect principally occurred in the cytoplasm, and the dual-vector tool could stably operate in HK-2 cells, increasing the translation level of the target gene FTH1. Therefore, the no NLS group dCasRx-eIF4GI translation enhancement tool was selected for the ensuing experiments.

### Application of the dual-vector dCasRx translation enhancement tool in a CaOx crystal-induced cell injury model

**Fig. 2:**
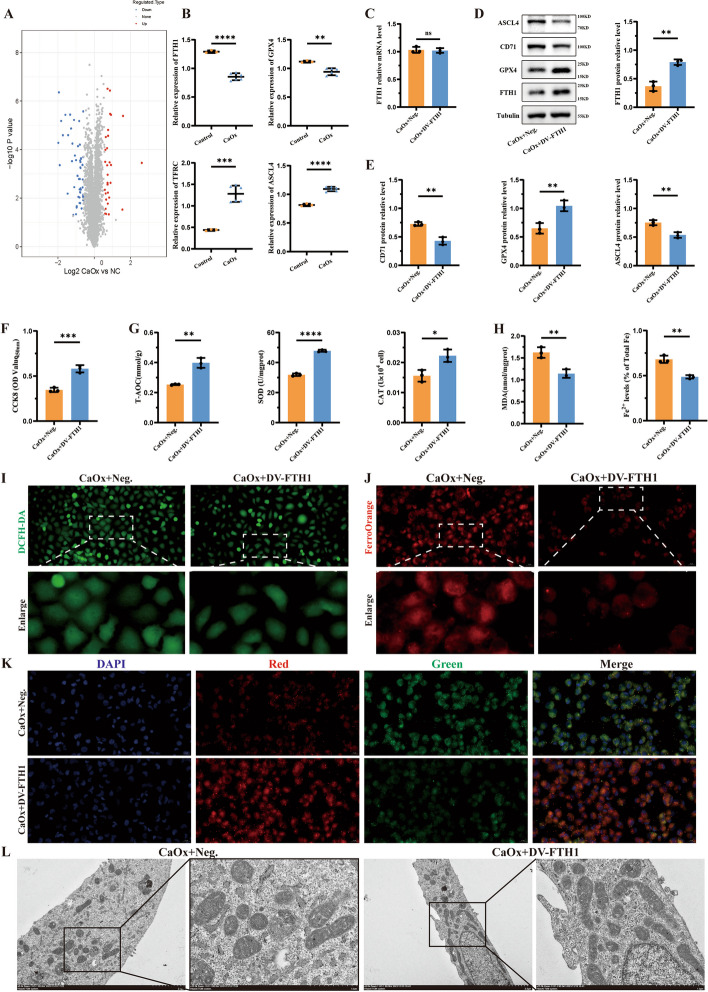
Application of the dual-vector dCasRx translation enhancement tool in a CaOx crystal-induced cell injury model. **A** Volcano plot shows differential expression genes (DEGs) between CaOx crystal-induced cell injury models and negative controls. **B** The protein expression of FTH1, GPX4, ASCL4, and CD71 (also called TFRC) in models were determined by 4D-LFQ proteomic analysis. **C** After constructing the dual-vector CRISPR-dCasRx-eIF4GI tool targeting FTH1 (DV-FTH1), the mRNA expression level of FTH1 were detected using qRT–PCR analysis. **D**, **E** WB analysis results show the protein expression levels of FTH1, CD71 GPX4, and ASCL4 between CaOx + Neg. and CaOx + DV-FTH1, and the bar graph shows the relative protein levels. **F** The cell viability was detected using CCK8 assay kit. **G** Several indicators of anti-oxidative status, including T-AOC content, SOD content and CAT content, were measured. **H** Two indicators of ferroptosis, including MDA content and Fe^2+^ content, were measured. **I** The level of cellular ROS was observed and images were taken under dark field (magnification ×400). Brighter green indicates higher levels of cellular ROS. **J** The level of cellular Fe^2+^ was observed and images were taken under dark field (magnification ×400). Brighter red indicates higher levels of cellular Fe^2+^. **K** The degree of lipid peroxidation was observed and images were taken under dark field (magnification ×400). Higher ratio of green to red indicates higher degree of lipid peroxidation. **L** The mitochondrial morphology was observed using transmission electron microscopy (scale bars, 2 μm or 1 μm). Data are presented as the means ± SEM from three independent experiments. *P < 0.05, **P < 0.01, ***P < 0.001, ****P < 0.0001, and ns, no significant difference, represents *P* > 0.05

To validate the potential application of the dCasRx translation enhancement tool, a renal stone cell model was established for subsequent validation. After stable transfection with the dual-vector translation enhancement tool, a crystal-induced cell injury model was constructed by treating HK-2 cells with CaOx crystals. As anticipated, the dual-vector dCasRx translation enhancement tool consistently exhibited efficiency postmodeling, almost unaffected by the disease model. Through 4D-LFQ proteomic analysis of differentially expressed proteins in the CaOx crystal-induced cell injury model, FTH1, TFRC (also called CD71), GPX4, and ACSL4 were selected for subsequent validation (Fig. 2A and B). Validation through RT–qPCR and western blotting revealed that, compared with the CaOx + Neg. (CaOx + Negative control) group, there was no significant difference in FTH1 mRNA expression levels (Fig. 2C), whereas FTH1 protein levels were significantly upregulated (Fig. 2D). Additionally, the protein levels of ACSL4 and CD71 were significantly downregulated, whereas GPX4 protein levels were significantly upregulated (Fig. 2E). Taken together, these results indicated that the dual-vector dCasRx translation enhancement tool could significantly upregulate the protein expression of the target gene FTH1 and indirectly modulate the expression levels of CD71, GPX4, and ACSL4 through the ferroptosis-related pathway. Following the translation enhancement of FTH1, cell viability was increased (Fig. 2F), and the levels of cellular antioxidant indicators, namely T-AOC, SOD and CAT, were significantly increased (Fig. 2G). On the other hand, its metabolites MDA and Fe^2+^ (Fig. 2H and J), the level of ROS (Fig. 2I), and the degree of intracellular lipid peroxidation (Fig. 2K) were significantly lower compared with the CaOx + Neg. group. Transmission electron microscopy illustrated alterations in mitochondrial morphology, with a substantial increase in the proportion of mitochondria and a significant decrease in solid and fragmented mitochondria in the group where FTH1 translation was enhanced using the translation enhancement tool (Fig. 2L). Our present results suggested that the dual-vector dCasRx translation enhancement tool could stably work in the CaOx crystal-induced cell injury model. Notably, enhancing the translation level of FTH1 significantly elevated the protein expression of FTH1, intracellular Fe^2+^ accumulation, cell antioxidant capacity, and mitochondrial function, thereby alleviating cellular lipid peroxidation and increasing cell viability. In summary, the dual-vector dCasRx translation enhancement tool inhibited ferroptosis and crystal-induced damage by upregulating the protein expression level of FTH1 in the CaOx crystal-induced cell injury model, hence playing a significant cellular protective role.

### Optimization design and preliminary functional validation of the single-vector dCasRx translation enhancement tool

**Fig. 3:**
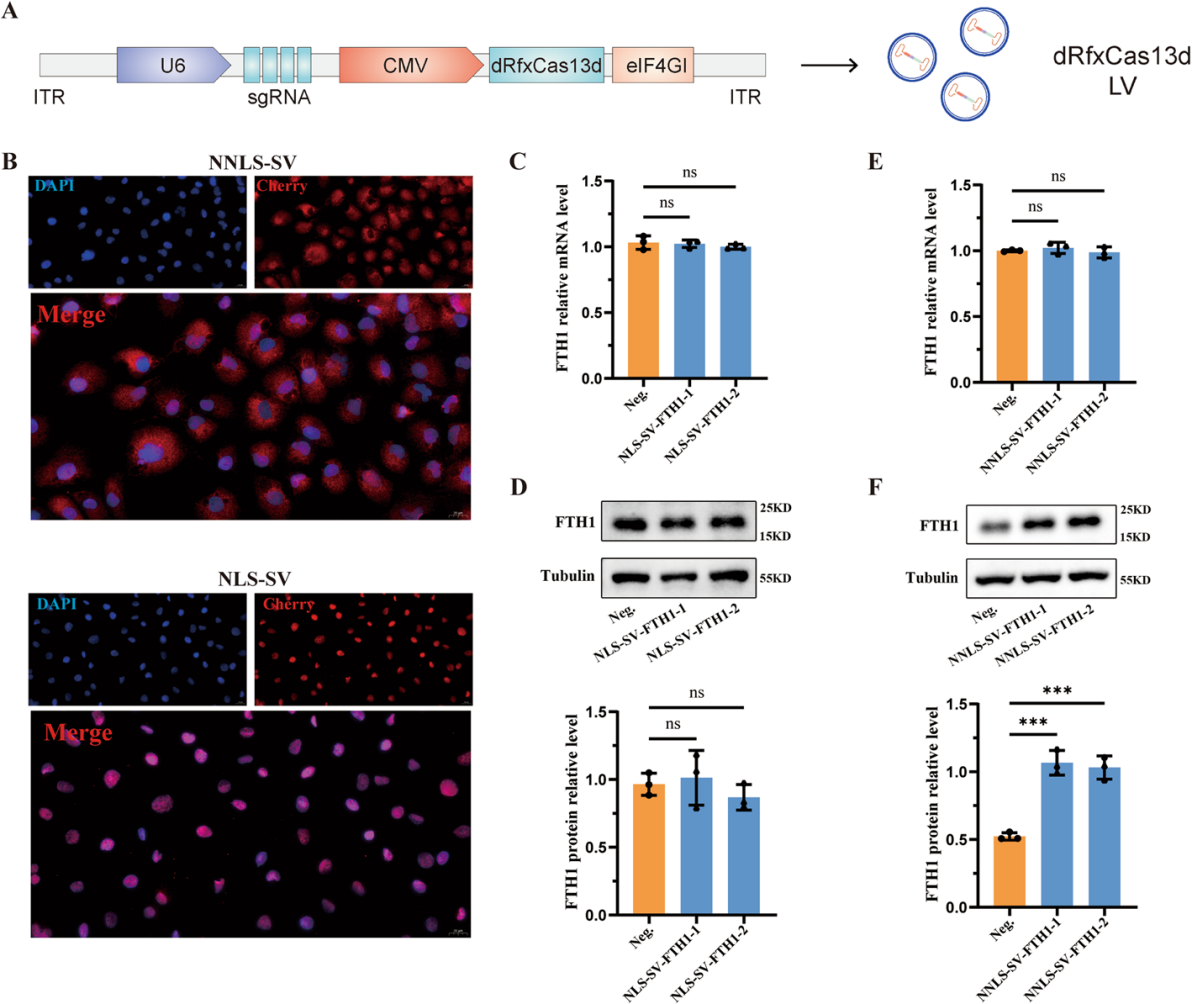
Preliminary design and functional validation of the single-vector dCasRx translation enhancement tool. **A** To package into lentiviral vectors, the dCasRx-eIF4GI fusion protein and its complementary DNA sequence, sgRNA, were cloned into plasmids containing both the CMV promoter and U6 promoter. **B** The subcellular localization of the single-vector dCasRx translation enhancement tool with/without NLS. Immunofluorescence were performed to further determine the localization of Cherry fluorescent label (magnification ×400). **C** After constructing the single-vector CRISPR-dCasRx-eIF4GI tool with NLS targeting FTH1 (NLS-SV-FTH1), the mRNA expression level of FTH1 were detected using qRT–PCR analysis. **D** The protein expression of FTH1 were verified using western blot and the bar graph shows the relative expression level of FTH1. **E** After constructing the single-vector CRISPR-dCasRx-eIF4GI tool without NLS targeting FTH1 (NNLS-SV-FTH1), the mRNA expression level of FTH1 were detected using qRT–PCR analysis. **F** The protein expression of FTH1 were verified using western blot and the bar graph shows the relative expression level of FTH1. Data are presented as the means ± SEM from three independent experiments. *P < 0.05, **P < 0.01, ***P < 0.001, ****P < 0.0001, and ns, no significant difference, represents *P* > 0.05

Our previous observation validated that the dual-vector dCasRx translation enhancement tool can stably work in HK-2 cells. However, the simultaneous cellular transfection of the dCasRx-eIF4GI fusion protein vector and sgRNA vector cannot ensure the consistency of their quantities entering the same cell, thereby impacting functional effects. Therefore, the design was optimized by constructing a single-vector LV tool to establish the more stable expression of the dCasRx translation enhancement tool, thereby enhancing its efficiency. Following the deletion of nonessential tool components, all necessary tool components were introduced into a single LV vector, driven by U6 and CMV promoters to express sgRNA and the dCasRx-eIF4GI fusion protein, respectively (Fig. 3A). To determine the subcellular functional localization of the single-vector dCasRx translation enhancement tool, Cherry fluorescence tags were fused to the tails of the dCasRx-eIF4GI fusion proteins of the NLS and no NLS groups, respectively. The fusion protein tools were then packaged into a single LV vector. sgRNA was designed for the target gene FTH1. After separate transfection into HK-2 cells, fluorescence localization was observed, exposing that the two sets of translation enhancement tools could be accurately localized in the nucleus or cytoplasm, depending on the presence of NLS tags (Fig. 3B). Thereafter, FTH1 mRNA and protein expression levels were detected using RT-qPCR and WB. Compared with the Neg. group, there was no significant difference in FTH1 mRNA levels in the NLS-SV-FTH1 group and NNLS-SV-FTH1 group after stable transfection with the dCasRx-eIF4GI-FTH1 single vector (Fig. 3C and E). On the other hand, after stable transfection with the dCasRx-eIF4GI-FTH1 single vector, the protein level of FTH1 in the NLS-SV-FTH1 group was comparable with that in the Neg. group (Fig. 3D). However, the protein level of FTH1 was significantly upregulated in the NNLS-SV-FTH1 group, compared with the Neg. group (Fig. 3F). These results corroborated that the no NLS group single-vector dCasRx translation enhancement tool and the no NLS group dual-vector dCasRx translation enhancement tool exhibited consistent subcellular localization, indicating their effective functionality in the cytoplasm.

### Universal applicability verification of the single-vector dCasRx translation enhancement tool

**Fig. 4:**
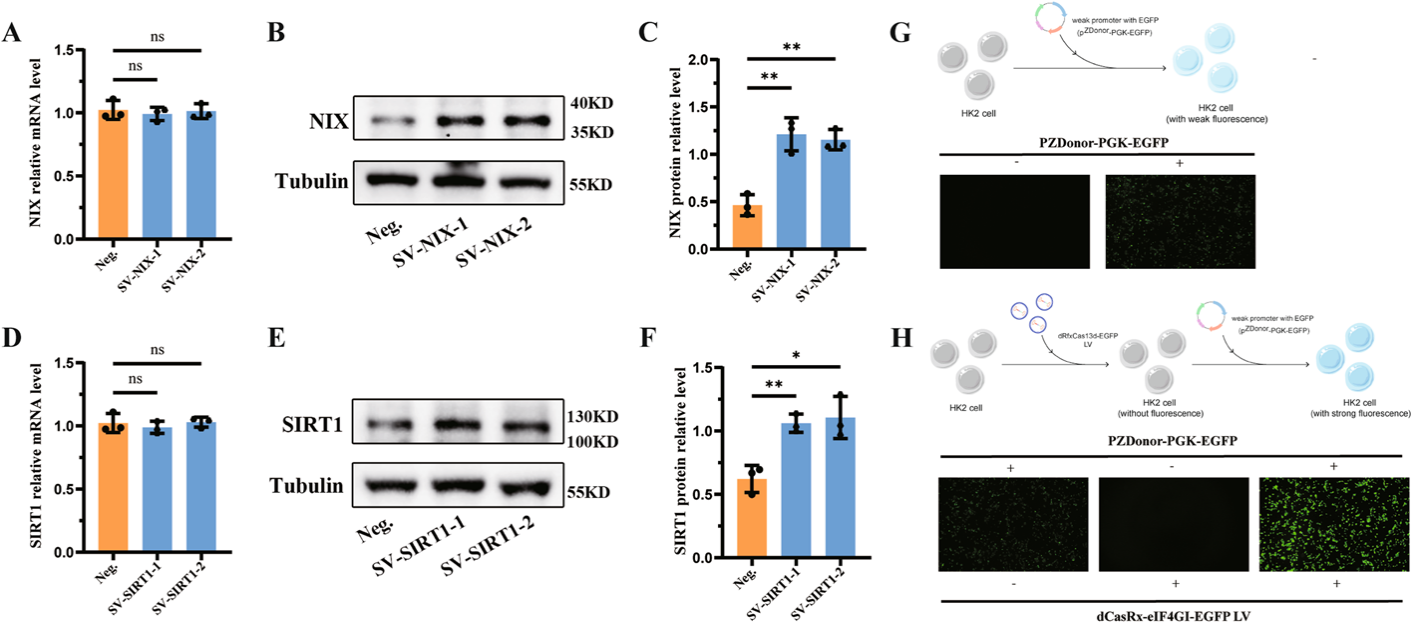
Universal verification of the single-vector dCasRx translation enhancement tool. **A** After constructing the single-vector CRISPR-dCasRx-eIF4GI tool targeting NIX (SV-NIX), the mRNA expression level of NIX was detected using qRT–PCR analysis. **B** The protein expression of NIX was verified using western blot and **C** the bar graph shows the relative expression level of NIX. **D** After constructing the single-vector CRISPR-dCasRx-eIF4GI tool targeting SIRT1 (SV-SIRT1), the mRNA expression level of SIRT1 was detected using qRT–PCR analysis. **E** The protein expression of SIRT1 was verified using western blot and **F** the bar graph shows the relative expression level of SIRT1. **G** After transfecting the plasmids containing the weak promoter with EGFP (pZDonor-PGK-EGFP) or negative control into HK2 cells for 8 h, respectively, the HK-2 cells were observed under an inverted fluorescence microscope and images were taken under dark field (magnification ×100). **H** After stably transfecting dCasRx-eIF4GI-EGFP into HK-2 cells, the plasmids containing the weak promoter with EGFP (pZDonor-PGK-EGFP) or negative control into HK2 cells for 8 h, respectively, the HK-2 cells were observed under an inverted fluorescence microscope and images were taken under dark field (magnification ×100). Data are presented as the means ± SEM from three independent experiments. *P < 0.05, **P < 0.01, ***P < 0.001, ****P < 0.0001, and ns, no significant difference, represents *P* > 0.05

To confirm the universal applicability of the single-vector dCasRx translation enhancement tool in mammalian cells, the two endogenous genes, namely SIRT1 and NIX, were selected. The former is a classical longevity gene that plays an antioxidant role in various kidney-related diseases, whilst the latter plays a critical role in the regulation of mitochondrial autophagy. After designing sgRNAs corresponding to NIX and SIRT1, the corresponding single-vector dCasRx translation enhancement tools were constructed. After stably expressing the translation enhancement tools in each group, qRT–PCR and WB were used to detect the mRNA and protein expression levels of the target genes. The results showed that, compared with the Neg. group, there was no significant change in FTH1, NIX, and SIRT1 mRNA levels in SV-NIX group and SV-SIRT1 group (Fig. 4A and D). Contrastingly, significant differences were observed in the protein levels of FTH1, NIX, and SIRT1 compared with the Neg. group (Fig. 4B, C, E and F). On the other hand, enhancing the translation level of exogenous genes further confirmed the universal applicability of the translation enhancement tool. EGFP is a classical exogenous fluorescent protein. Plasmids with a weak promoter driving EGFP (pZDonor-PGK-EGFP) were then transfected into HK-2 cells along with negative control plasmids. After transfecting for 8 h, HK-2 cells transfected pZDonor-PGK-EGFP exhibited weak fluorescence expression, whereas the negative control group displayed no fluorescence expression. (Fig. 4G). Then, the corresponding sgRNA sequences were designed for pZDonor-PGK-EGFP and we constructed the single-vector dCasRx-eIF4GI-EGFP translation enhancement tool. After stably transfecting dCasRx-eIF4GI-EGFP into HK-2 cells, similarly, pZDonor PGK-EGFP plasmids were then transfected into HK-2 cells along with negative control plasmids. After 8 h, the fluorescence intensity was determined, revealing a significant increase in fluorescence intensity in HK-2 cells transfected with both dCasRx-eIF4GI-EGFP translation enhancement tool and pZDonor PGK-EGFP plasmids (Fig. 4H). This finding confirmed that the single-vector dCasRx translation enhancement tool still exerted its translation enhancement function at the mammalian cell level when targeting exogenous genes. In short, intervention at the translation level for both endogenous and exogenous genes unveiled that the single-vector dCasRx translation enhancement tool had favorable universal applicability and promising development prospects.

### Evaluation of the efficiency of the single-vector dCasRx translation regulation tool

**Fig. 5:**
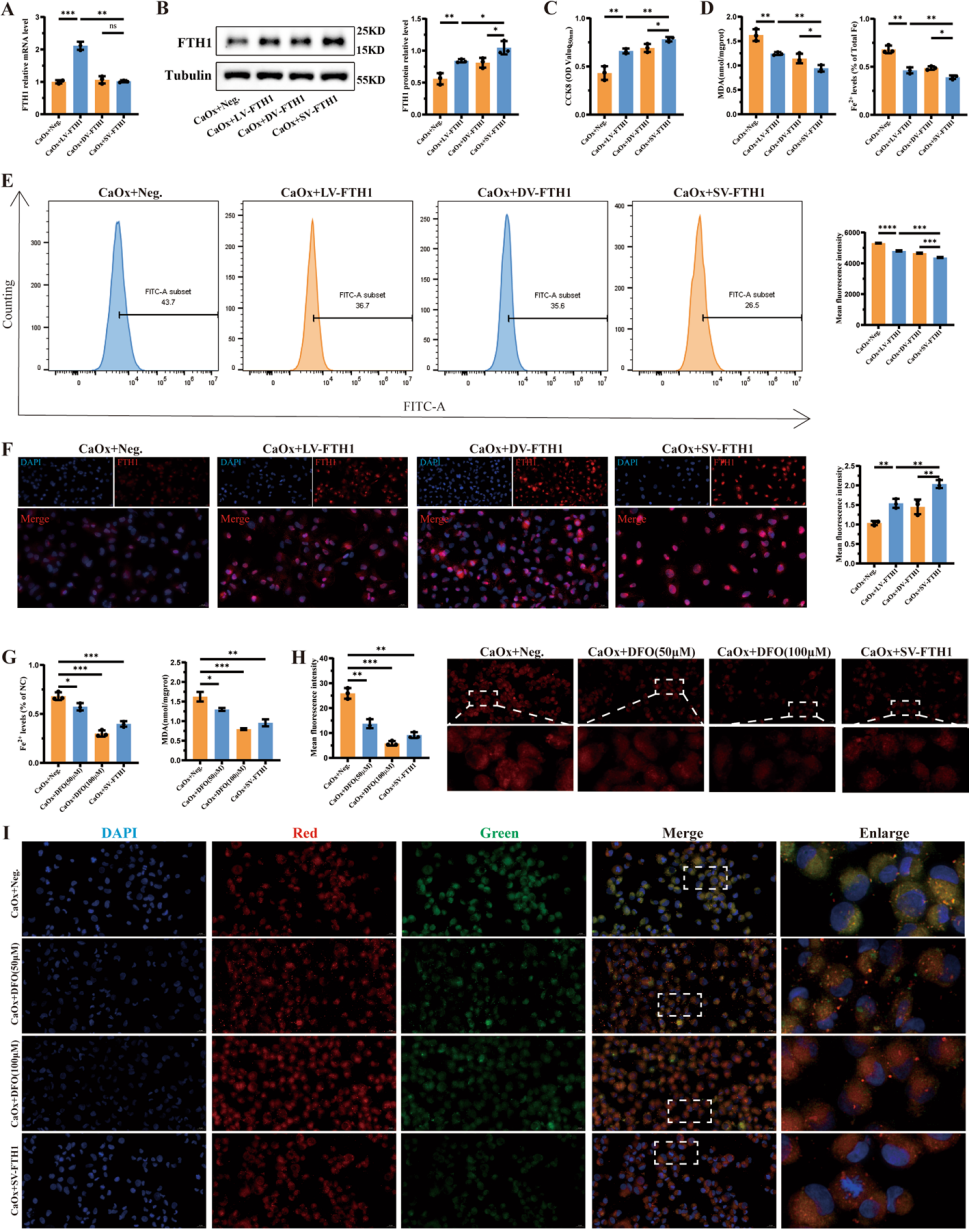
Application of the single-vector dCasRx translation enhancement tool in a CaOx crystal-induced cell injury model. The HK2 cell lines were stably transfected with lentiviral vectors for FTH1 overexpression (LV-FTH1), the dual vector CRISPR-dCasRx-eIF4GI tool targeting FTH1 (DV-FTH1), the single-vector CRISPR-dCasRx-eIF4GI tool targeting FTH1 (SV-FTH1). **A** The mRNA expression level of FTH1 was detected using qRT–PCR analysis for four groups. **B** The protein expression of FTH1 was verified using western blot and the bar graph shows the relative expression level of FTH1. **C** The cell viability was detected by CCK8 assay kit. **D** Two indicators of ferroptosis, including MDA content and Fe^2+^ content, were measured. **E** The degree of lipid peroxidation was measured using the flow cytometric analysis in four groups and the bar graph shows the mean fluorescence intensity. **F** Immunofluorescence analysis was performed to determine the expression of FTH1 (magnification ×400) and the bar graph shows relative protein levels. **G** Deferoxamine (DFO) is an iron chelator and a ferroptosis inhibitor. The 50 μM and 100 μM concentration of DFO is used. Two indicators of ferroptosis, including Fe^2+^ content and MDA content, were measured. **H** The level of cellular Fe^2+^ was observed and images were taken under dark field (magnification ×400). Brighter red indicates higher levels of cellular Fe^2+^ and the bar graph shows the mean fluorescence intensity. **I** The degree of lipid peroxidation was observed and images were taken under dark field (magnification ×400). Higher ratio of green to red indicates higher degree of lipid peroxidation. Data are presented as the means ± SEM from three independent experiments. *P < 0.05, **P < 0.01, ***P < 0.001, ****P < 0.0001, and ns, no significant difference, represents *P* > 0.05

After stable transfection of HK-2 cells with the single-vector dCasRx translation regulation tool, its protein expression regulation efficiency was compared with that of conventional LV vector gene overexpression tools and the dual-vector dCasRx translation regulation tool. qRT–PCR and western blotting were employed to detect the expression levels of FTH1 mRNA and protein, respectively. The results revealed that, compared with the two CRISPR-dCasRx-based translation enhancement tools, the stable expression of the conventional LV vector gene overexpression tool led to a significant increase in intracellular FTH1 mRNA levels (Fig. 5A). After stable expression of the single-vector dCasRx translation regulation tool, there was significantly increasing in FTH1 protein expression levels compared with the other three groups, thereby verifying the aforementioned findings (Fig. 5B and F). Then, the cell viability was further reversed in CaOx + SV-FTH1 group (Fig. 5C). Compared with the other three groups, stable transfection and expression of the single-vector dCasRx translation regulation tool resulted in a significant reduction in ferroptosis metabolites, namely MDA and Fe^2+^ (Fig. 5D), and the levels of the intracellular lipid peroxidation (Fig. 5E). In conclusion, the single-vector dCasRx translation enhancement tool acts as a more effective translation activation method for the target gene FTH1 through performing more stable intracellular expression. In comparison with conventional gene overexpression tools and dual-vector translation enhancement tools, the single-vector dCasRx translation enhancement tool had better performance in elevating the protein expression level of the target gene FTH1, leading to a significant improvement in the inhibition of ferroptosis and cellular protection. Therefore, the single-vector dCasRx translation enhancement tool was selected for the following experiments. Additionally, deferoxamine (DFO), an iron chelator, can reduce iron accumulation to inhibit ferroptosis [28]. Following the addition of DFO (50 μM and 100 μM), the levels of Fe^2+^ and MDA (Fig. 5G and H), and the degree of lipid peroxidation has a significant decrease (Fig. 5I).

### Application of the single-vector dCasRx translation enhancement tool in a mouse kidney stone model

**Fig. 6:**
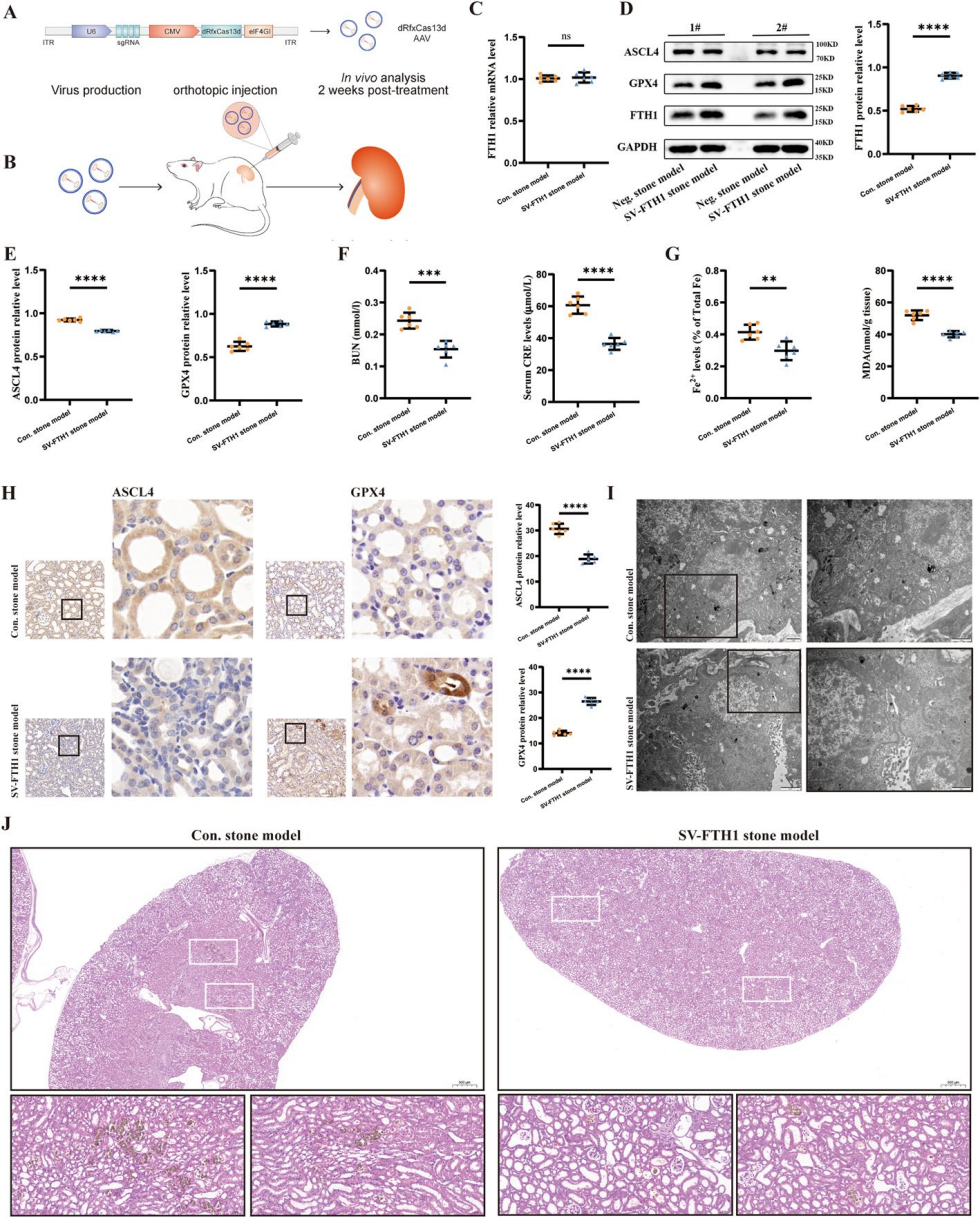
Application of the single-vector dCasRx translation enhancement tool in a mouse kidney stone model. **A** To package into adenoassociated viral vectors, the dCasRx-eIF4GI fusion protein and sgRNA complementary to DNA sequence of related genes was inserted into the consent skeleton containing the CMV and U6 promoters to form the plasmid dCasRx-eIF4GI-sgRNA. **B** Flow chart of animal model construction. **C** The mRNA expression level of FTH1 were detected using qRT–PCR analysis between Neg. stone model and SV-FTH1 stone model. **D**, **E** The protein expression of FTH1, ASCL4, and GPX4 were verified using western blot and the bar graph shows the relative expression levels. **F** Two indicators of kidney injury, including BUN content and serum CRE content, were measured. **G** Two indicators of ferroptosis, including Fe^2+^ content and MDA content, were measured. **H** The images show the degree of renal expression for GPX4 and ASCL4 in two groups (magnification ×400) and bar graph shows relative protein levels. **I** The mitochondrial morphology alterations associated with ferroptosis were observed using transmission electron microscopy (scale bar,  μm or 1 μm). **J** Crystal deposition was observed. Data are presented as the means ± SEM from six independent experiments. *P < 0.05, **P < 0.01, ***P < 0.001, ****P < 0.0001, and ns, no significant difference, represents *P* > 0.05

To evaluate the in vivo efficiency of the single-vector dCasRx translation enhancement tool, a single-vector AAV translation enhancement tool was designed and constructed according to the aforestated vector construction strategy (Fig. 6A). Following the injection of AAV into mouse kidneys in situ and verification of the successful expression of the single-vector AAV translation enhancement tool, the mouse kidney stone disease model was established, and samples were harvested (Fig. 6B). Noteworthily, the single-vector AAV translation enhancement tool was also stably expressed in the kidneys of mouse kidney stone model mice and exerted corresponding effects. Afterward, RT-qPCR, WB analysis, and immunohistochemical experiments demonstrated that the single-vector AAV translation enhancement tool could stably elevate the translation of the target gene FTH1 without impacting FTH1 mRNA transcription (Fig. 6C), thereby significantly increasing the expression level of the FTH1 protein and indirectly regulating the protein expression of ACSL4 and GPX4 (Fig. 6E and 6H). Furthermore, the levels of MDA and Fe^2+^ (Fig. 6G) in renal tissue were significantly lower in the SV-FTH1 stone model group compared with the Con. stone model group (stone model alone group), with renal function displaying a moderate degree of recovery (Fig. 6F). The projection electron microscopy was used to visualize fluctuations in mitochondrial morphology in kidney tissues. The results showed that the proportion of mitochondria was significantly higher in the SV-FTH1 stone model group than in the Con. stone model group, whilst the degree of mitochondrial shrinkage and fragmentation was significantly lower than in the Con. stone model group (Fig. 6I). Moreover, after stable performing of the single-vector AAV translation enhancement tool designed for FTH1, it could effectively attenuate the degree of ferroptosis in mouse kidney stone model and alleviate kidney damage. Finally, calcium staining displayed that the Con. stone model group exhibited a higher degree of calcium crystal deposition in kidney tissues. Conversely, the SV-FTH1 stone model group, maintaining a stable expression of the translation regulation tool, possessed a significantly decreased calcium crystal deposition in the kidneys of the mouse kidney stone model (Fig. 6J).

## Discussion

Gene regulation strategies within the CRISPR system related to CRISPR–Cas systems involve the fusion of numerous functional factors used to modulate transcription or perform epigenetic modifications on RNA/DNA [10, 29, 30]. Research on the regulation of protein translation is currently scarce despite being crucial for controlling gene expression at the translational level. While effective strategies are available for manipulating gene expression at the genomic or transcriptional levels, methods for translation regulation are scarce [8, 31]. Considering the mRNA translation process is the final step in gene expression and directly influences the corresponding gene expression, thus manipulating translation regulation may offer advantages in studying gene expression levels. Hence, there is an urgent need to develop and construct a translation regulation tool platform to target mRNAs.

Effector proteins of class 2 CRISPR–Cas systems are typically composed of a single large multidomain Cas protein [5, 6], showing superior developmental potential. In class 2 CRISPR–Cas systems, DNA editing tools such as CRISPR–Cas9 have a well-established technical platform and are universally used in various bioengineering and molecular therapeutic strategies [32]. Although Cas9 systems have been optimized to recognize and act on single-stranded RNA by introducing a short DNA oligonucleotide segment binding to the target RNA sequence, tools for RNA editing and manipulation based on the CRISPR–Cas9 technology platform lag behind DNA-related tools due to technical limitations. A critical limitation in RNA editing and manipulation is the paucity of effective and specific targeting and introduction of RNA-binding domains into target cells, restricting strategies for studying RNA-related functions. Through bioinformatics analysis of prokaryotic and eukaryotic genomic sequences, a class of CRISPR–Cas systems that specifically target RNA was identified and named type VI CRISPR systems, specifically CRISPR–Cas13d. The Cas13d ribonuclease exhibits highly specific targeting and negligible off-target effects. Among the Cas13d family, CasRx is a smaller effector with a highly specific cleavage function. The catalytically inactive dCasRx effector can efficiently bind to RNA without altering mRNA and protein levels, making dCasRx fusion proteins a flexible RNA-binding module for targeting specific RNA sequences.

In eukaryotic cells, eIF4G forms the translation initiation complex eIF4F by binding with eIF4A and eIF4E, which subsequently activates the translation initiation process. eIF4GI is the dominant form of eIF4G in mammalian cells [4], and the fusion protein of eIF4GI can activate translation by enhancing ribosome recruitment without influencing mRNA levels, providing operability within mammalian cells. In the present study, the CRISPR-dCasRx effector was connected with the functional element of eIF4GI to enhance the translation of target mRNAs in target cells through specific sgRNA guidance. Analysis of a cell model of CaOx kidney stones using 4D-LFQ quantitative proteomics highlighted significant differences in the expression levels of the ferroptosis-related proteins FTH1, ACSL4, GPX4, and CD71 in the CaOx stone model group compared with the control group. Meanwhile, the autophagic degradation of FTH1 can facilitate ferroptosis via iron overload, making it a crucial inhibitory gene for ferroptosis [26]. Prior studies have concluded that inhibiting the degradation of FTH1 in the stone model group significantly reduced Fe^2+^ levels in HK-2 cells and intracellular ferroptosis levels and effectively inhibited cell-crystal damage. Therefore, FTH1 was selected as the target gene for subsequent verification experiments. Given that the biological process of translation essentially occurs in the cytoplasm, two types of dual-vector translation enhancement tools were developed for subcellular localization: nuclear entry group (NLS) and nonnuclear entry group (NNLS). Analysis of the mRNA and protein expression levels of FTH1 uncovered that the NLS group did not work after stable transfection, while the NNLS group efficiently worked after stable transfection. The NNLS group dual-vector dCasRx translation regulation system, following introduction into mammalian cells, did not influence the mRNA levels of the target gene but only played a role in enhancing translation, increasing the protein expression intensity of the corresponding gene.

Furthermore, the in vitro and in vivo kidney stone disease model was constructed by stabilizing and enhancing the expression of FTH1 in HK-2 cells through the CRISPR-dCasRx-eIF4GI system. After FTH1 overexpression using this translation enhancement system, the expression of positively regulated ferroptosis proteins CD71/ACSL4 was significantly downregulated, and that of the negatively regulated ferroptosis protein GPX4 was significantly upregulated, compared with the CaOx + Neg. group. Moreover, intracellular Fe^2+^ levels, lipid peroxidation, and ROS levels were markedly decreased, whereas cell activity was significantly increased. The overall ferroptosis level in cells was significantly inhibited, demonstrating a significant protective effect against cell-crystal damage. To further improve the efficiency of the translation enhancement tool and clarify the subcellular localization of the tool, redundant label proteins were discarded by optimizing the fusion protein structure. The fusion functional elements and sgRNA were inserted into a single LV vector, and following the addition of a fluorescent label, the NNLS group single-vector dCasRx translation regulation system could be located within the cytoplasm of HK-2 cells and work efficiently. To validate the universal applicability of the single-vector dCasRx translation enhancement tool, several sgRNAs corresponding to endogenous proteins (e.g., FTH1/SIRT1/NIX) were designed. The results showed a significantly elevated expression intensity of the above proteins. Indeed, the single-vector dCasRx translation enhancement tool demonstrated wide applicability in enhancing the translation of various endogenous proteins in mammalian cells. Following the construction of the kidney stone disease model using HK-2 cells stably expressing CRISPR-dCasRx-eIF4GI-FTH1, the stable enhancement of the expression intensity of the target gene FTH1 significantly downregulated the positively regulated ferroptosis proteins CD71/ACSL4 and upregulated the negatively regulated ferroptosis protein GPX4. Besides, intracellular Fe^2+^ levels, lipid peroxidation, and ROS levels were markedly decreased, whereas cell activity was significantly increased. The overall intracellularly ferroptosis level was significantly suppressed, attenuating the degree of cell-crystal damage. Moreover, the FTH1 overexpression effect of the single-vector translation enhancement system significantly outperformed traditional overexpression LV tools and dual-vector CRISPR-dCasRx-eIF4GI-FTH1, providing a more effective inhibition of ferroptosis. In the mouse kidney stone disease model constructed by stably expressing CRISPR-dCasRx-eIF4GI-FTH1, the trend of differentially expressed proteins and ferroptosis-related indicators was consistent with the cell model. It is worthwhile emphasizing that the tool mitigated kidney damage in the stone model and minimized the degree of crystal deposition in kidney tissues by stably enhancing the expression intensity of the target gene FTH1, thereby significantly inhibiting the formation of kidney stones. It can be deduced that the single-vector dCasRx translation enhancement tool can perform stably and efficiently in disease models and has promising prospects for targeted improvements in kidney stone models.

Translation initiation is a rate-limiting step in the regulation of translation [1]. Herein, the dCasRx was connected with the eIF4GI functional element. After the target mRNA bound to dCasRx through sgRNA recognition, eIF4GI stimulated cap-dependent translation initiation, activated translation by enriching ribosomes, and enhanced protein translation levels. In previous studies, CRISPR-mediated transcriptional activation significantly increased the mRNA expression levels of target genes, upregulating the corresponding protein expression. However, the mRNA levels of genes do not always linearly correlate with their corresponding protein expression levels, and translation is the final step in gene expression that determines expression intensity [13, 33]. Compared with traditional tools, the dCasRx translation enhancement tool offers several advantages: (1) it is the smallest effector in the Cas13 family, contributing to packaging and delivery in a single vector while offering outstanding modification potential; (2) it can stably express and efficiently enhance the translation levels of multiple endogenous genes in mammalian cells without impacting the genetic information of the host genome; and (3) the upregulation in the target gene’s expression intensity is significantly superior to that of traditional tools. In various kidney-related diseases, an imbalance in the translation levels of certain key proteins can promote the development of the disease [34]. Over a hundred therapeutic proteins have been utilized in various molecular therapeutic strategies, and the regulation of protein translation urgently warrants further research [35]. In this study, the dCasRx translation enhancement tool was used to upregulate the translation initiation rate of the target gene FTH1, significantly increasing its expression intensity. This further showcased the inhibitory effect of FTH1 on stone formation, and the translation enhancement tool was successfully applied to a kidney stone disease model. Its overexpression effect on FTH1 was significantly higher than traditional technology platforms and did not affect FTH1 mRNA levels, effectively inhibiting intracellular Fe^2+^ and ferroptosis levels. Furthermore, it significantly protected cells from oxidative stress damage, demonstrating working efficacy and promising application prospects in a disease environment. At the same time, based on previous reports on ferroptosis-related research, most agonists (e.g., erastin, RSL3, etc.) and inhibitors (e.g., Fer-1) that modulate ferroptosis levels mostly mediate ferroptosis levels by activating or inhibiting lipid peroxidation levels [22]. Few studies have reported intervention agents or tools specifically targeting iron or Fe^2+^ accumulation [27]. This study proposed a molecular biological strategy based on the dCasRx translation enhancement system that can selectively increase the translation level of FTH1, significantly attenuating ferroptosis-induced Fe^2+^ overload and cell damage. It also provides a selective molecular biology strategy for regulating Fe^2+^ accumulation in ferroptosis research.

## Conclusions

The dCasRx translation enhancement system and the translation enhancement operation targeting FTH1 hold considerable potential for practical applications. It not only provides novel insights and a universal platform for translation regulation research but also offers a selective molecular biology strategy for regulating Fe^2+^ accumulation, making molecular therapeutic strategies for kidney stones a promising option for future clinical treatment.

### Supplementary Information

**Additional file 1.** Supplementary Table S1.

**Additional file 2.** Supplementary Table S2.

**Additional file 3.** Supplementary Table S3.

## Data Availability

The datasets used and/or analyzed during this study are available from the corresponding author on reasonable request.

## Abbreviations

CRISPR: Clustered Regularly Interspaced Short Palindromic Repeats
CasRx/ RfxCas13d: Cas13d family ribonuclease effector
dCasRx: Deactivated Cas13d family ribonuclease effector
eIF4G: Eukaryotic translation initiation factor 4G
eIF4E: Cap-binding protein
eIF4A: RNA helicase
eIF3: Eukaryotic translation initiation factor 3
CaOx: Calcium oxalate
5' UTR: 5' Untranslated region
IRES: Internal ribosomal entry site
LV: Lentivirus
AAV: Adeno-associated virus
NLS: Nuclear localization signal
LFQ: Label Free Quantitation
FTH1: Ferritin Heavy Chain
TFRC/CD71: Transferrin Receptor
GPX4: Glutathione Peroxidase 4
ASCL4: Acyl-CoA synthetase long chain family member 4
NIX: BCL2/adenovirus E1B interacting protein 3-like
SIRT1: Sirtuin 1
T-AOC: Total antioxidant capacity
SOD: Superoxide dismutase
CAT: Catalase
EGFP: Enhanced green fluorescent protein

## References

[1] Tahmasebi S, Khoutorsky A, Mathews MB, Sonenberg N (2018). Translation deregulation in human disease. Nat Rev Mol Cell Biol.

[2] Das S, Das B (2016). eIF4G-an integrator of mRNA metabolism?. FEMS Yeast Res.

[3] Gradi A, Imataka H, Svitkin YV, Rom E, Raught B, Morino S (1998). A novel functional human eukaryotic translation initiation factor 4G. Mol Cell Biol.

[4] Bovee ML, Lamphear BJ, Rhoads RE, Lloyd RE (1998). Direct cleavage of elF4G by poliovirus 2A protease is inefficient in vitro. Virology.

[5] Koonin EV, Makarova KS, Zhang F (2017). Diversity, classification and evolution of CRISPR-Cas systems. Curr Opin Microbiol.

[6] Makarova KS, Wolf YI, Alkhnbashi OS, Costa F, Shah SA, Saunders SJ (2015). An updated evolutionary classification of CRISPR-Cas systems. Nat Rev Microbiol.

[7] Makarova KS, Zhang F, Koonin EV (2017). SnapShot: class 2 CRISPR-Cas systems. Cell.

[8] Klann TS, Black JB, Chellappan M, Safi A, Song L, Hilton IB (2017). CRISPR-Cas9 epigenome editing enables high-throughput screening for functional regulatory elements in the human genome. Nat Biotechnol.

[9] Chavez A, Scheiman J, Vora S, Pruitt BW, Tuttle M, Eswar PRI (2015). Highly efficient Cas9-mediated transcriptional programming. Nat Methods.

[10] Konermann S, Lotfy P, Brideau NJ, Oki J, Shokhirev MN, Hsu PD (2018). Transcriptome engineering with RNA-targeting type VI-D CRISPR effectors. Cell.

[11] Wessels HH, Méndez-Mancilla A, Guo X, Legut M, Daniloski Z, Sanjana NE (2020). Massively parallel Cas13 screens reveal principles for guide RNA design. Nat Biotechnol.

[12] Granados-Riveron JT, Aquino-Jarquin G (2018). CRISPR-Cas13 precision transcriptome engineering in cancer. Cancer Res.

[13] Liu Y, Beyer A, Aebersold R (2016). On the dependency of cellular protein levels on mRNA abundance. Cell.

[14] Yang SX, Song C, Xiong YH (2020). Current perspectives on urolithiasis management in China. World J Urol.

[15] Dong C, Song C, He Z, Liao W, Song Q, Xiong Y (2023). An overview of global research landscape in etiology of urolithiasis based on bibliometric analysis. Urolithiasis.

[16] Faller N, Dhayat NA, Fuster DG (2019). Nephrolithiasis secondary to inherited defects in the thick ascending loop of henle and connecting tubules. Urolithiasis.

[17] Hoppe B, Martin-Higueras C (2020). Inherited conditions resulting in nephrolithiasis. Curr Opin Pediatr.

[18] Ye Z, Zeng G, Yang H, Li J, Tang K, Wang G (2020). The status and characteristics of urinary stone composition in China. BJU Int.

[19] Amato M, Lusini ML, Nelli F (2004). Epidemiology of nephrolithiasis today. Urol Int.

[20] Dong C, Zhou J, Su X, He Z, Song Q, Song C (2024). Understanding formation processes of calcareous nephrolithiasis in renal interstitium and tubule lumen. J Cell Mol Med.

[21] Khan SR, Canales BK, Dominguez-Gutierrez PR (2021). Randall's plaque and calcium oxalate stone formation: role for immunity and inflammation. Nat Rev Nephrol.

[22] He Z, Liao W, Song Q, Li B, Liu J, Xiong Y (2021). Role of ferroptosis induced by a high concentration of calcium oxalate in the formation and development of urolithiasis. Int J Mol Med.

[23] Forcina GC, Dixon SJ (2019). GPX4 at the crossroads of lipid homeostasis and ferroptosis. Proteomics.

[24] Muhoberac BB, Vidal R (2019). Iron, ferritin, hereditary ferritinopathy, and neurodegeneration. Front Neurosci.

[25] Ohgami RS, Campagna DR, McDonald A, Fleming MD (2006). The Steap proteins are metalloreductases. Blood.

[26] Gao M, Monian P, Pan Q, Zhang W, Xiang J, Jiang X (2016). Ferroptosis is an autophagic cell death process. Cell Res.

[27] Li J, Cao F, Yin HL, Huang ZJ, Lin ZT, Mao N (2020). Ferroptosis: past, present and future. Cell Death Dis.

[28] Bruzzese A, Martino EA, Mendicino F, Lucia E, Olivito V, Bova C (2023). Iron chelation therapy. Eur J Haematol.

[29] Cao C, Yao L, Li A, Zhang Q, Zhang Z, Wang X (2021). A CRISPR/dCasX-mediated transcriptional programming system for inhibiting the progression of bladder cancer cells by repressing c-MYC or activating TP53. Clin Transl Med.

[30] Li J, Chen Z, Chen F, Xie G, Ling Y, Peng Y (2020). Targeted mRNA demethylation using an engineered dCas13b-ALKBH5 fusion protein. Nucleic Acids Res.

[31] Xu X, Qi LS (2019). A CRISPR-dCas toolbox for genetic engineering and synthetic biology. J Mol Biol.

[32] Doudna JA, Charpentier E (2014). Genome editing. The new frontier of genome engineering with CRISPR-Cas9. Science.

[33] Greenbaum D, Colangelo C, Williams K, Gerstein M (2003). Comparing protein abundance and mRNA expression levels on a genomic scale. Genome Biol.

[34] Schaeffer V, Abrass CK (2010). Mechanisms and control of protein translation in the kidney. Am J Nephrol.

[35] Leader B, Baca QJ, Golan DE (2008). Protein therapeutics: a summary and pharmacological classification. Nat Rev Drug Discov.

